# Utilization of Internet of Things and Wireless Sensor Networks for Sustainable Smallholder Agriculture

**DOI:** 10.3390/s22093273

**Published:** 2022-04-24

**Authors:** Amsale Zelalem Bayih, Javier Morales, Yaregal Assabie, Rolf A. de By

**Affiliations:** 1 Department of Geo-Information Processing, Faculty of Geo-Information Science and Earth Observation (ITC), University of Twente, P.O. Box 217, 7500 AA Enschede, The Netherlands; j.morales@utwente.nl (J.M.); r.a.deby@utwente.nl (R.A.d.B.); 2 Department of Computer Science, College of Natural and Computational Sciences, Addis Ababa University, Addis Ababa P.O. Box 1176, Ethiopia; yaregal.assabie@aau.edu.et

**Keywords:** Internet of Things, wireless sensor network, affordable digital data infrastructure, technology assist in smallholder data acquisition, smart agriculture

## Abstract

Agriculture is the economy’s backbone for most developing countries. Most of these countries suffer from insufficient agricultural production. The availability of real-time, reliable and farm-specific information may significantly contribute to more sufficient and sustained production. Typically, such information is usually fragmented and often does fit one-on-one with the farm or farm plot. Automated, precise and affordable data collection and dissemination tools are vital to bring such information to these levels. The tools must address details of spatial and temporal variability. The Internet of Things (IoT) and wireless sensor networks (WSNs) are useful technology in this respect. This paper investigates the usability of IoT and WSN for smallholder agriculture applications. An in-depth qualitative and quantitative analysis of relevant work over the past decade was conducted. We explore the type and purpose of agricultural parameters, study and describe available resources, needed skills and technological requirements that allow sustained deployment of IoT and WSN technology. Our findings reveal significant gaps in utilization of the technology in the context of smallholder farm practices caused by social, economic, infrastructural and technological barriers. We also identify a significant future opportunity to design and implement affordable and reliable data acquisition tools and frameworks, with a possible integration of citizen science.

## 1. Introduction

Agriculture contributes more than 25% to the gross domestic product (GDP) [1] and 55% to employment opportunities in developing countries [2]. It is thus a prominent sector to target poverty reduction, and is crucial for economic global policy-making. Smallholder farming forms a significant component of this sector with a food production rate of more than 70% globally and more than 60% in developing countries [3]. For instance, in Ethiopia, smallholder farming accounts for about 95% of total agricultural production of the country [4]. An estimated 2.5 billion people practice smallholder agriculture and, in doing so, manage about 510 million farms [5].

Smallholder farmers often own a fragment of farmland and operate under perilous conditions due to their heavy reliance on ecosystem services that can be scarce and potentially show signs of degradation [6,7]. Unpredictable and extreme weather conditions may bring drought or flooding, which lead to soil erosion and depletion, hindering farms from operating at full potential [8]. The limited financial capacity of smallholder farmers also means that their investments in yield-improving mechanisms are restricted. As a result, an area of focus in these communities is an affordable means to increase farm production with efficient and sustained utilization of available resources [6].

The development of context-specific solutions is critical to improve and sustain better production by smallholders. It is important to invest in agricultural practices that can, not only provide for future generations, but are also climate-resilient [9,10]. Such approach requires a data richness that comes directly from the farm allowing informed decision-making on issues such as how to prepare land, what to plant, how much to consume and how much to sell or store [11]. Real-time information on farming systems, soil and crop health, water management, livestock management, and agro-climatic attributes such as rainfall and soil moisture could enable smallholder farmers to practice more sustainably and increase their productivity.

Sustainable agriculture consists of practices that opt to meet present and future food demands while fulfilling social and economic equity, protecting environmental health and increasing profitability [9,12,13]. Sustainable agriculture and optimization of farm yield needs better understanding of local and farm level contexts of farm production and productivity parameters, of which some are variable in space and over time. At the same time, most smallholder farms are found in rural areas, which often display poor infrastructure and sites are often inaccessible, challenging the collection of these data. As a result, information produced is rarely complete and often does not quite fit with farm levels. Even when data are available it is commonly outdated and not representative of current situations. Consequently, intuitive farming decisions and traditional farm practices persist among associated communities and cause over-exploitation of the scarce resources leading to poor production [14]. Thus, viable technological approaches that are easily adoptable and reliable are needed for the efficient acquisition of in situ farm data. With proper enabling tools, such data can be obtained affordably [11].

Recently, with the advent of technology and powerful computing infrastructures, it has become possible to obtain, analyze, and generate sound agricultural information in a cost-effective and timely manner. Remote sensing (RS), proximal sensing (PS) and in-field sensing (IfS) paved the way for enhanced data acquisition complementing conventional approaches such as survey and physical field observation [15,16]. A detailed discussion of each approach is presented in Section 3. The large spatial and temporal coverage and recent advances in high-resolution imagery, has made satellite technology indispensable to capture farm-related data remotely. Recent works have shown how RS platforms like Sentinel-1/-2 and MODIS can be used to efficiently capture farm data [17,18,19]. PS technology, with more closer look than RS, is effectively utilized for better farm observation, agricultural disaster management, and agricultural land classification [20,21]. IfS with spatially dispersed network of small, inexpensive, and efficient sensing devices, known as wireless sensor networks (WSN) and the Internet of Things (IoT) has created opportunities for precise farm monitoring [22,23].

Various authors have reported the effective utilization of WSN and IoT for soil data collection in large-scale farming [24,25,26,27]. It remains unclear how effective the case can be in smallholder agriculture. Thus, this paper aims to establish scientific understanding concerning:

1. Whether IoT and WSN can also be used and potentially benefit smallholder farming
2. Whether the immediate data needs of the smallholder farmer are addressed, and
3. Which design and implementation issues need to be considered for IoT and WSN application in smallholder agriculture.

To this effect, we analyze literature on the state-of-the-art over the past ten years, 2011–2021. The analysis is done through a comprehensive literature review and presented in sections below. The paper summarizes prior research work, explores key limitations and prospects, and identifies knowledge gaps. This work also proposes a high-level, WSN-based IoT architecture for agricultural application and discusses the challenges and opportunities related to IoT implementation in smallholder agriculture. Published journals, white papers, conference proceedings and real systems are the inputs to our work.

**Figure 1:**
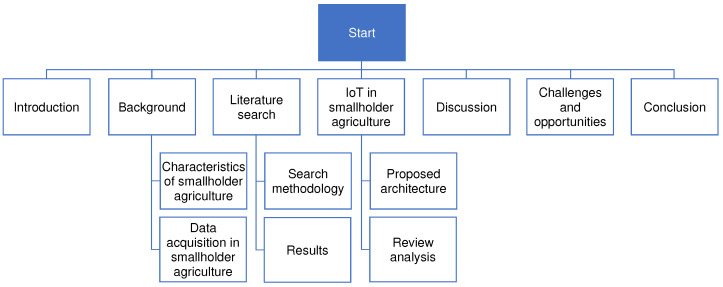
Schematic workflow representation of the proposed work.

The remainder of this paper is organized as follows. Section 2 describes the unique characteristics of smallholder farms and the main factors affecting their productivity. Section 3 discusses relevant approaches to data collection in smallholder agriculture. With these, we aim to layout a better insight of the target population and bring about the data gaps that needs to be filled. The literature search methodology is explained in Section 4. An in-depth analysis of the use of IoT and WSN in smallholder agriculture, based on reviewed literature and a high-level system architecture are presented in Section 5. Discussion and possible opportunities and challenges are discussed in Section 6 and Section 7, respectively. Section 8 presents our conclusions. The overall workflow of the paper is shown in Figure 1.

## 2. Characteristics of Smallholder Agriculture

Smallholder farmers are often characterized as being conservative, tech-resistant and showing a low adoption rate to innovations [28]. It is important to understand the root causes for these which may lie in the community. With this knowledge, we may avoid the incongruity between technology adoption and actual farmers’ conditions. Certain farm or farming characteristics are common amongst smallholders and distinguish them from large-scale enterprise farming. Almost by definition, land size is a key property. Based on this, smallholder farms are those practiced on a small portion of land, 2 ha on average [7,29]. Most smallholder farms are also known to practice farming on the basis of natural biophysical conditions to crop growth, such as rain [29], and to apply a mix of crop growing and livestock breeding. However, the extent of these characteristics may vary with demographic and economic conditions of the location, the ecological zone and other terrain parameters [7]. A systematic, multi-criterion definition needs to be formulated from similar characteristics exhibited by the farmers. These characteristics can be categorized in four dimensions:

- Social attitude;
- The use of resources and infrastructure;
- Awareness and preparedness;
- Productivity.

### 2.1. Social Attitude

Family ownership, subsistence farming, risk avoidance, communal information needs and reliance on extension services are social characteristics of smallholder farms. Most smallholder farms are run by a family and mostly for the family’s subsistence with small production [7]. The condition of total dependence on the farm to feed the family creates attitudes of considerable risk avoidance and resistance to costly farming practices with farmers. Consequently, there is slow adoption of technology and new farming approaches [28]. Instead, low-return but sustained production practice is preferred over technology-supported farming by most smallholders [7].

Some forms of information provide added value when shared amongst members of the farmer community. Adjacent plots owned by different smallholders will often be under similar farming practices and communication between neighbor farmers is more readily trusted. As a result, relevant information emerging in the community may get communal use. Agricultural extension workers also assist farmers through community level knowledge transfer regarding improved farm management, input application and other tasks towards better productivity. They serve as a bridge to pass on higher level decisions to farmers and oversee their implementation. This makes smallholder farms highly dependent on extension workers for information and know-how.

### 2.2. Resource Usage and Infrastructure

Small farm size, farming practices with mixed crops, limited access to finance and weak transport, communication and on-farm infrastructure are conditions familiar to most smallholder farms. A farm’s land may be further split into smaller but discontinuous plots where different crops are cultivated. This practice expresses a risk mitigation mechanism to stabilize farm income and reduce the impact of failure shocks, even when it is reported to have negative correlation with yields [30]. Diverse cropping on fragmented lands causes complications for technological assistance and innovation, for one because plot and crop requirements may vary. The required investments make farmers resistant to such changes, which are better suited to and have often been developed for larger-scale farming.

Their limited financial capacity also makes farmers less interested in technological investments. They heavily rely on farming and have minimal or no alternative source of income [7]. Most farms are remote and suffer from poor physical and institutional infrastructure in provision of electric power, road access, irrigation, communication networks, and access to social services [31]. These challenges hinder successful implementation of agricultural innovation.

### 2.3. Awareness and Preparedness

In the absence of agricultural information systems, smallholders are vulnerable to natural hazards. Water excess or scarcity are particularly dangerous because most farms apply rain-fed practices. Adoption of agricultural innovation and technology is hard to achieve without systematic assistance and training. In addition, the generally low literacy rate of smallholders causes low awareness and preparedness for on-farm solutions. This requires attention to be paid to minimal initial investment level, ease of use, sensitivity to culture, language and other acceptance barriers. Fuelled by scientific finds, it is crucial to expand rural education and regular capacity building of extension workers.

### 2.4. Low Productivity

Various socio-economic, environmental and biophysical factors often correlate strongly with production levels of smallholders. They include genetically determined behavior of crops, soil properties, climate conditions, topography, farm financial status and illiteracy. Weak land management practices [32], the volume of family labor [33] and uninformed use of farm inputs are also factors that contribute to poor farm productivity. The low productivity is also often exacerbated by crop disease and pests [34,35]. In addition to the inculcated effect of the above factors, scarce research and development activities and lack of accurate and timely information negatively impact farm yields [36,37].

It is worth noting that these factors do not equally impact farming across all smallholder landscapes. Their role and severity vary with micro-climate, region, and agro-ecological zone. Understanding them as root causes within the space-time variability is critical for finding contextual solutions. Our review, as discussed in Section 5.2, reveals soil (in its various properties) as the most central attribute that impacts agricultural production. We should thus strive to obtain accurate on-farm information about relevant soil properties to allow valuable diagnostics.

Considering the aforementioned characteristics, smallholder farms can be defined as: *family-run, small farms that are often fragmented into disjoint plots, that are nature-dependent and have limited operational budget, limited information access and technology support, and often display low production levels*.

## 3. Data Acquisition in Smallholder Agriculture

Comprehensive data on farm inputs, natural resource utilization, and environmental and biophysical attributes is required to assist in improving farming. There are four possible ways to acquire both technical and socio-economic data at the smallholder farm level. These are: conventional, or by remote sensing, proximal sensing and in-field sensing.

- **Conventional data collection** Interviews, focus group discussions, surveys, sample collections and observations, on-farm trials and yield measurements are the conventional primary data collection approaches used in agronomic research and projects [38,39,40]. These are techniques for which physical presence of the data collector is needed, and they demand high resource mobilization, especially human labor. Sample collection, on-farm trials, observations and measurements are used to collect physical data such as soil properties and fertility, crop condition and yield estimates, while interviews, focus group discussions and surveys can be used for socio-economic data collection. In general, these data collection techniques exhibit a number of challenges. (i) Prolonged collection period to capture precisely spatio-temporal variability [41]; (ii) non-traceability as most smallholders have little formal education and do not keep written records of parameters during farm work, leading to lack of evidence; (iii) data collection is considered intrusive and less-trusted by farmers as the activities are mostly non-participatory and thus less transparent [40]. Collected data may be biased due to misinformed collectors or the use of poor sampling schemes. This may result in incomplete data with quality problems, subsequently leading to incorrect conclusions. However, with careful and proper execution, conventional tools are crucial for calibration and other statistical analytic purposes [38]. Such data may also complement and/or validate secondary data used in decision-making systems.
- **Remote sensing** Recent research efforts have shown the potential of RS and GIS technologies for monitoring smallholder farms and filling data gaps [42,43]. RS uses space-borne and airborne systems and generates valuable data such as crop phenotype, growth stages and crop health issues, soil type, soil moisture and farm input uses [44]. The potential of RS to assess crop and soil properties, and farm inputs is demonstrated in [17,19,43,45,46]. The actual application of these tools is uncommon in developing countries. The lack of ground truth data, the high learning curve to understand and use the outcomes, the coarse spectral resolution as well as lack of appropriate training are reasons for their limited use. Small and fragmented farms, vegetation heterogeneity, and atmospheric conditions also diminish quality and usefulness of RS data [47,48]. Nonetheless, RS has high potential in complementing conventional data collection approaches and holds promise to assist sustainable farming practices.
- **Proximal sensing** A closer look at a farm field can be achieved with PS; sensors positioned approx. 2 m above the field surface then capture data [49]. A number of agricultural parameters including soil properties, crop growth metrics and farm inputs can be acquired through this platform [50,51].This is done by electromagnetic sensors, ground penetrating radar (GPR) and gamma-ray spectrometry sensors [15,47]. PS sensors are typically mounted on tractors, spreaders, sprayers or irrigation booms. PS technology is relatively economical, more accurate and offers higher spectral resolution than RS [52]. However, low temporal resolution, labor intensity and significant cost hinder full usability. The scarcity or absence of mechanized farming vehicles is another barrier to utilization of PS. A review of both RS and PS technology in soil data collection and associated challenges is provided in [53].
- **In-field sensing** IoT and WSN can complement the above approaches and help minimize their mentioned challenges. IoT is a platform through which objects can be interconnected to generate and exchange relevant and valuable information. Objects can be both physical and virtual. WSN is at the heart of this platform enabling reliable interaction between dispersedly located objects. IoT and WSN have application in the broader agricultural domain. Monitoring, tracking, automation, and precision agriculture can be mentioned in this context. Several projects are known in the developed world: the EU’s Food and Farm 2020 project, the Kansas water preservation through sensors, and NanoGanesh are a few examples [54]. The NanoGanesh project, for instance, is an irrigation automation system that creates mobile-based remote control for water pumps and water tanks using sensor information. The project helps farmers to control water pumps, their power supply, and provides vandalism alerts of field-deployed equipment. Other projects have designed affordable, all-inclusive farm improvement mechanisms that utilize IoT and WSNs [25,26,55,56]. Smallholder farmers require information and advice, down-scaled to the plot level, to improve their practice; stakeholders such as agricultural extension agents, and (non-)governmental aid organizations also rely on such information for planning. The point-level data offered through IoT and WSN makes the technology fitting with only a limited number of sensors per plot. Inexpensive sensors keep things affordable, also to smallholders. In addition, IoT and WSN are simple and quick approaches for farm-level data collection that allow direct interpretation. Further discussion of IoT and WSN is presented in Section 5.

## 4. Literature Search Methodology

Agriculture encompasses various activities including crop farming and livestock breeding. IoT and WSN can have various applications in this broad domain including farm monitoring, animal tracking, and precision agriculture. Our literature search considered all these sub-domains and followed the Preferred Reporting Items for Systematic Reviews and Meta-Analyses (PRISMA) framework as a standard [57]. The use of IoT and WSN for agricultural applications was insignificant until around 2010 and has increased significantly since then. Hence, we searched for publications in the years 2011–2021. Research articles, software and data publications, white papers and conference proceedings were searched and acquired from three citation databases: ACM, Scopus and ScienceDirect. Our search query and inclusion and exclusion criteria are presented in Table 1.

**Figure 2:**
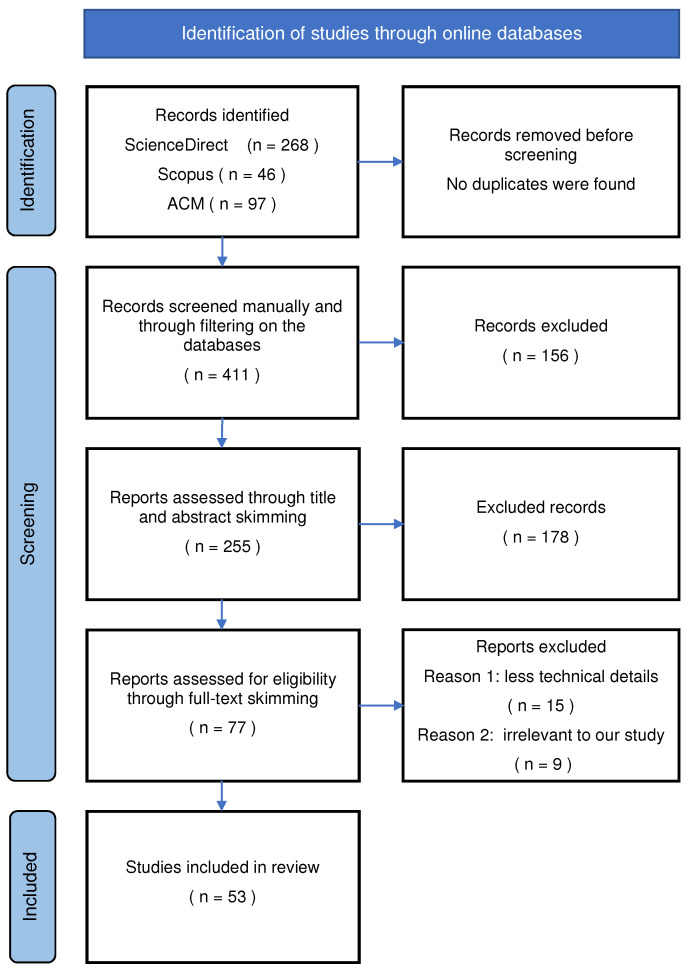
Search procedures for review papers and obtained results based on PRISMA framework.

Our searches followed a three-step approach: paper acquisition, paper filtering and paper analysis. Before paper acquisition, a metadata search was executed on the citation databases. Reviews, early access articles, theoretical frameworks, editorials, encyclopedia and magazines were excluded. Publications that addressed large-scale enterprise farms or deployment in resource-rich environments were also excluded. Thirdly, publications with little or unclear technical discussion or with little focus on the physical or hardware details were left out from our review. Figure 2 shows the schematic representation of our search results.

A first round search was conducted between February and March 2021, and a second round in December 2021. A crude total of 411 papers from more than 20 journals were identified. Next, results were filtered using the exclusion criteria mentioned in Table 1. A first level discrimination was based on the paper’s type, followed by title and abstract skimming to assess whether the scope of the work falls into our research interest. Finally, full-text read was conducted to check the relevance, technicality, usability and deployment environment.

Some 53 papers were found relevant to our study, of which about 50% were in conference proceedings. Further analysis was carried out on these papers to understand the hardware and technical details. Sensors and nodes used in the work, communication technologies, power sources and energy harvesting schemes, micro-controllers and transceivers were specifically studied. Section 5 presents a detailed discussion of findings.

## 5. IoT and WSN in Smallholder Agriculture

**Figure 3:**
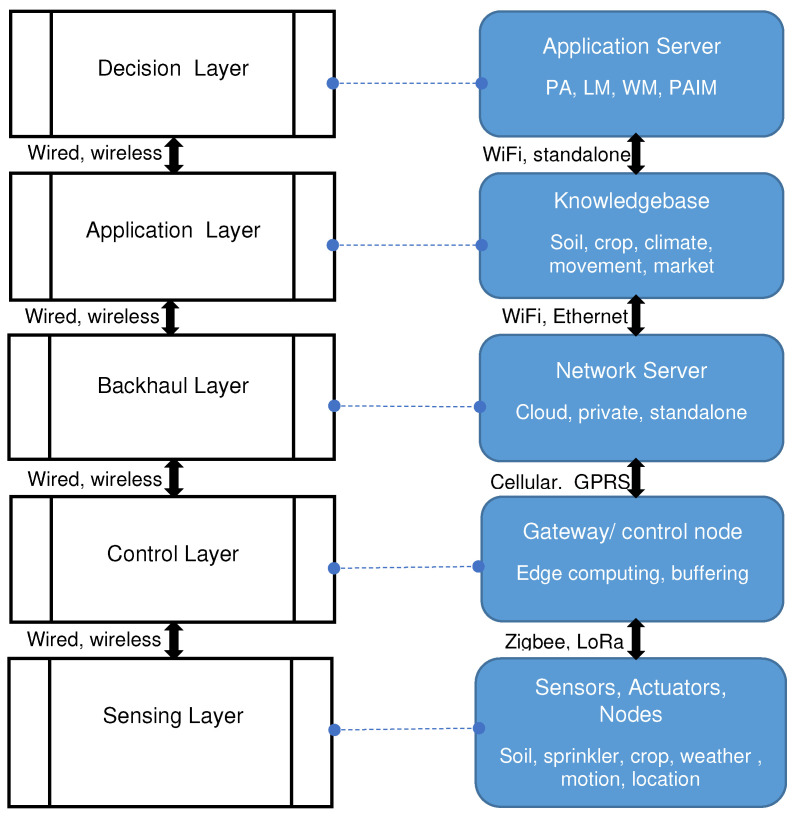
A general Internet of Things (IoT)-wireless Sensor Network (WSN) architecture for agriculture applications (PA:Precision Agriculture, LM: Livestock Management, WM: Weather Monitoring, PAIM: Pest and Animal Infestation Monitoring).

We argued in Section 3 that IoT has sound justification to assist in agriculture data collection and address the information gap that smallholder farms face. Monitoring crops, soil and weather at farm-level, continually and in (near) real-time with reasonable cost may provide closer insight into possible farm investments for improved productivity. Livestock tracking, pest detection, farm input application may support decision-making and better farm management practice.Unfortunately the deployment areas where IoT is most useful are also those the technology is most vulnerable to theft, vandalism and other physical damages. Social awareness campaigns can help prevent this and enhance the capability of a community to collect data that assist its agricultural practices. With proper design and implementation considerations, smallholder farms can be made to benefit from the technology. In line with this, We propose an IoT-WSN architecture for agricultural applications. The proposed architecture is presented in Figure 3 and it has five layers: sensing, control, backhaul, application and decision layer.

As can be seen in the figure, the *sensing layer* comprises objects used to monitor and interact with agricultural elements such as soil, livestock or crop. Measurement sensors and their controlling nodes are found on this layer. Nodes run embedded instruction on when to measure which agriculture elements through one or more sensors. Data collected from this layer needs to reach the control layer through wired or wireless communication protocols. Data rate, power consumption and communication range are some important factors that affect the choice. Less power-hungry and optimal communication are required in agriculture applications. Examples of wireless communication protocols are presented in Table 2. In real agricultural fields’ deployment, communication signal could be weak due to environmental obstructions such as tree. This might cause frequent communication interruption between sensing layer and the control layer with significant data loss. Design and/or implementation considerations such as obstruction-aware deployment scheme or node’s temporary data buffering scheme are required. The *control layer* is responsible for aggregation of received data and transfer to the back end system. It also controls actions of actuators through decisions coming from the back end system. Based on the device’s capability and application requirement, this layer can be setup to perform additional tasks such as controlling the frequency and time of data reading by sensor nodes and data buffering. A node with higher processing and communication capability is setup at this layer. This node is often referred as gateway and supports different communication protocols. The *backhaul layer* bridges the communication between the resource-constrained gateway device and relatively resource-rich back end system. It manages the underlying network and application communication protocols between gateway and network server and between the network server and application server. In the later case, the communication protocols can be synchronous as in Message Queuing Telemetry Transport (MQTT) or asynchronous as in Constrained Application Protocol (CoAP). The network communication protocol can be the reliable Transport Control Protocol (TCP) or the efficient User Datagram Protocol (UDP). In both cases, the protocol choice depends on energy-efficiency, inter-operability and resource need. It is possible for this communication to be disrupted due to unstable communication infrastructure. Alternatives that could withstand such disruptions may need to be identified. The *application layer* defines the business rules and required functionality of the agriculture application. This layer also creates the required seamless integration of data coming from heterogeneous sensors. Finally, the *decision layer* performs analysis of data received from devices based on the business rules and user requirements. Data analysis, report generation and decision support all happen in this layer. The type of information that is generated, target stakeholders, information re/presentation and delivery interface are all decisions that must be addressed at the level of this layer. The application and decision layers shall address requirements of a specific agriculture application domain and answer all users’ needs. This trickles down and dictate the type and number of nodes to deploy at the sensing layer as well as the timing and amount of data to read. Usefulness of decisions made at these two layers also heavily rely on the quality of data generated at lower layers. Hence, data validation and quality assurance measures needs to be in place at the lower layers.

Actual installation and operation of IoT is often challenged in smallholder communities due to poor infrastructure and high improvement cost. More cost-effective and less resource-hungry solutions such as WSN are potential candidates for integration with the IoT [107]. Essentially, the low-power wireless communications contribute to improving both IoT device connections with the Internet and the overall efficiency of the IoT application operation. Important types of wireless communication technology are presented in Table 2. Minimal operational resource needs, scalability, long data range, high resistance to signal interference, simple operation and maintenance requirements are critical criteria when choosing a wireless communication technology for such environments.

Our analysis reveals that use of IoT and WSN in smallholder farming evolves around the following issues:

- Purpose;
- Sensor deployment and implementation;
- Communication technology;
- Power sources;
- Computing analysis;
- Quality assurance.

We elaborate on these below.

### 5.1. Purpose

Agricultural productivity depends on multiple parameters: those of soil, weather, crop and other farm management practices especially. Careful investigation is, thus, required to identify those parameters with most impact, to allow tuning farming practices through continuous monitoring and analysis.

Four areas have been associated by the research community with the use of IoT and WSN in smallholder agriculture: precision agriculture (PA), weather monitoring (WM), pest and animal infestation monitoring (PAIM) and livestock management (LM). In *precision agriculture*, work focuses on automatic irrigation, fertilization and other farm input management actions. PA aims at precise and efficient use of agricultural inputs for optimal production. The precision addresses dosage, application location or timing. Irrigation management, using monitored soil data from sensors, and deploying actuators contributes to efficient and timely water utilization [59,62,65,66,68,69,73,77,90,99,103,108,109,110]. Monitoring of soil nutrients and soil moisture content helps control fertilizer application and promotes the optimal use of other natural resources [67,75,87,98,101,111].

In areas with poor coverage, *Weather monitoring* can potentially benefit from IoT and WSNs through sensor-based micro-climate weather stations [70,82,83,89,94,105,112]. Weather data by itself does not fully predict crop performance because other variables are also in play. The correct approach is to integrate with other agro-climatic variables [67,88,105,113]. Correlation between soil moisture, air temperature and humidity, and other environmental variables needs to be analyzed for agricultural applications. Existing agro-meteorological data collection infrastructures in smallholder context are at best sparse. This is mainly because of budget constraints. As a result, the quality and quantity of produced data are poor [83,94]. Setting up automatic weather stations through WSN may complement these weather stations. In addition to being cost-effective, they can be deployed densely to produce data of better spatial resolution, and can be configured to acquire data autonomously at small time intervals. This brings value in agricultural early warning systems.

The third area in which IoT and WSN is used in smallholder agriculture is *pest and animal infestation monitoring*. Crop pests and diseases and unwanted animal visits to farmlands are known causes of crop loss. Both wild and domestic animals pose such threats and farmers can benefit from real-time monitoring of animal visits to fields. Knowing a crop’s resource requirements at the different growth stages and timely observation of leave color changes may reduce crop failure caused by pests and diseases. Temperature and motion sensors can capture data relevant to these problems. IoT and WSN can help to set up a sensor network cost-effectively and to the required precision and temporal scale [60,61,81,92,93,114,115].

*Livestock management* is the fourth area that may benefit from IoT and WSN technology [79,80,86,104,116,117,118,119]. Most smallholders practice mixed crop farming in combination with livestock breeding. The livestock may be as important as the crops. It is mostly an income source but it can also be a source for the household’s dietary needs. Tracking of cattle, monitoring animal health and safety of feedlots and barns, and monitoring body changes can become sensor-based.

**Figure 4:**
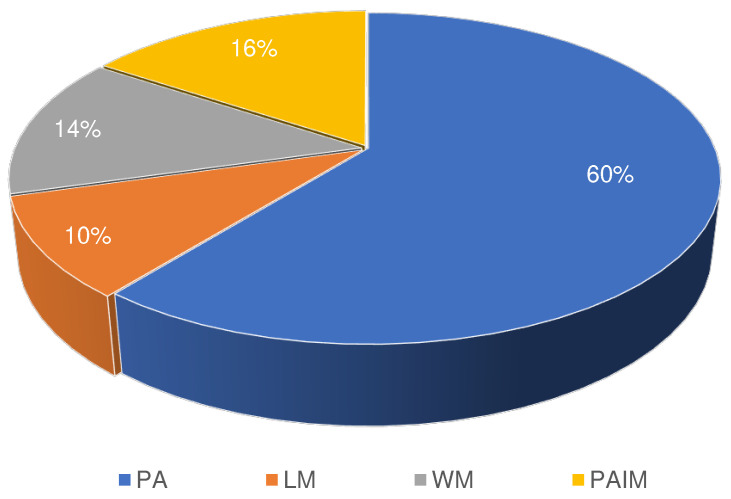
Thematic distribution of reviewed papers (N=53) depicting important uses of IoT-WSN in smallholder agriculture (PA:Precision Agriculture, LM: Livestock Management, WM: Weather Monitoring, PAIM: Pest and Animal Infestation Monitoring); classes may be mutually overlapping.

Among the 53 papers reviewed, we found that PA is researched widely with about 64% of the works targeting it, as shown in Figure 4. The classes in that figure are not mutually exclusive as some papers address more than one application domain, as discussed in Section 5.1 and reported in [91,99]. Most data collected was used in water management and irrigation. Since smallholder farms mostly apply rain-fed practices, other use cases that address the much needed information of the community shall be sought. These can be deciding which crop to plant, the sowing time and pest management [98,120,121]. Weather sensor data may also help in situations like drought. Precise information on such and optimal utilization of other existing farm resources is, thus, also of paramount importance to this community.

### 5.2. Sensor Deployment Schemes

Multiple sensors make up a WSN. Sensor deployment in agriculture requires a careful design that takes into account the spatio-temporal variability of the parameters of interest. For instance, soil moisture may display high spatial and temporal variability while crop growth tends to be slow and monotonic over time, and has higher predictability. Appropriate sensor deployment shall address such variability. It also determines the number of devices to be used, with budget implications. Moreover, some agricultural parameters, like soil moisture and crop root growth, exhibit spatial variability over different soil depths. Optimal placement of sensors is required to address such requirements and optimization of performance and coverage of the WSN [111].

Work by [72] presents WSN deployment from three perspectives: node movement (dynamic, static, hybrid), node type (homogeneous, heterogeneous) and system hierarchy (single-tier, multi-tier). In static node deployment, nodes are placed in a fixed position for the entire sensing duration [122]. The dynamic node set-up allows mobility of nodes, and a hybrid set-up integrates static and dynamic nodes. Homogeneous nodes are those with near-identical characteristics while heterogeneous nodes differ in computation power, sensing capability and/or transceiver units. A tiered hierarchy organizes nodes into clusters to extend the spatial coverage with the same [123] or a different [122] number of nodes in each cluster. Sensor placement in WSN typically follows a random [122] or grid-based approach [75,106,111]. In the first, nodes are distributed randomly over the target location. In a grid-based set-up, nodes are arranged in a regular equidistant way, placed at the edge or center of the polygon [106,124,125]. As most reviewed papers are experimental, little is discussed regarding node placement. A few papers mention cluster-based static sensor placement [75,106], and in livestock monitoring, nodes are tied to moving animals.

**Figure 5:**
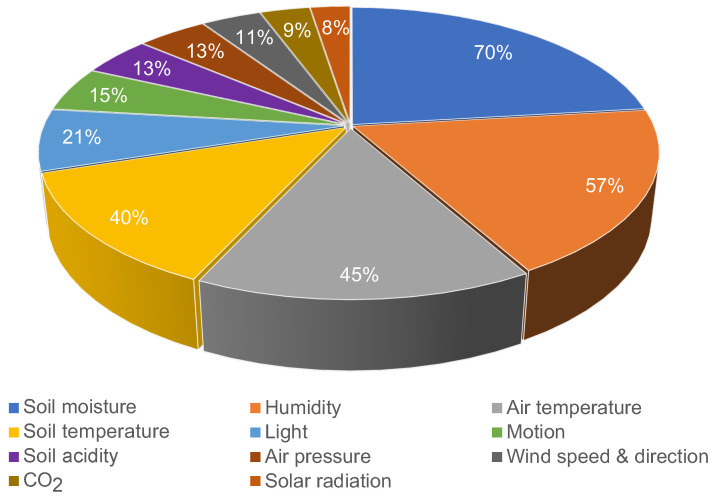
Sensor type distribution of reviewed papers (N=53) showing important types in smallholder agriculture. Papers typically report multiple sensor types in use.

The number and type of sensors in a WSN for agriculture monitoring are specific to the application theme or purpose. Sparse air temperature and humidity sensors may suffice to collect weather data at village-level. High sensor density is needed with motion sensors in livestock monitoring, unless the purpose is to follow group movements only. Measurements from some sensors can also serve multiple needs. For instance, relative humidity can be used for weather analysis and in crop pest manifestation assessment. A dense deployment of nodes with high frequency data collection generates a large amount of data. However, such affects the network’s lifetime and comes with budget implications. The papers we reviewed use different sensor types. In PA applications, soil water content or soil temperature measurement are common while in livestock management animal movement is required. Some sensor types are also commonly found among applications: soil moisture and relative humidity sensors are frequently used, as shown in Figure 5. Air and soil temperature sensors are also often deployed sensors while others like light, CO${}_{2}$, location, soil electrical conductivity (EC), soil acidity (pH), wind speed and direction are less often applied [79,80,82,83,86,92,94,101,110,111,118,126,127].

Most agricultural parameters require time-series monitoring. Expected parametric temporal variability is the key factor to determine sensor reading frequency and period; in principle, one wants to capture each significant parameter value change. An additional concern is especially the daily cycle in the sensor’s ambient conditions, which may negatively impact comparability of sensor readings. Thus, sensors should collect data at fixed moments in the day, where it needs to be understood that ‘at 7 a.m.’ and ‘one hour after sunrise’ are different notions of time fixation. Still, post-processing of sensor data should analyze whether the day/night rhythm is manifest in the data, and how, if so, to correct for it.

In PA, for instance, as plants grow their demand for resources evolves. It may thus be beneficial to monitor throughout the season for optimal production. In pest monitoring, the appropriate sensing period may fall between the flowering and senescence phases. For some parameters, even within the active sensing window, measurements are required more frequently as, for instance, in soil moisture compared to soil type [128]. In most reviewed papers, a data read frequency may range between 15 and 30 min with no specific justification. We observed more emphasis given to the energy use efficiency of nodes and less focus to the efficient applicability of acquired data.

As discussed in Section 2, smallholder farms have communal data needs and similar characteristics in weather and soil attributes. Thus, it is more cost-effective to deploy a variety of sensors than a dense scatter of redundant sensors to obtain a more holistic view. A WSN node can listen to multiple sensors as most reviewed papers actually do. This helps to improve understanding of site-specific interplay between parameters. It also simplifies communication management when more nodes are deployed. Overall, an efficient sensor deployment scheme for smallholder agriculture shall give due consideration to wide area coverage, efficient power use, extended network lifetime, reduced signal loss, low cost and reliable data generation. It is also crucial to acquire all needed data to the required spatial-temporal resolution. These are discussed in less detail in the reviewed papers. Only some projects mention a systematic node deployment strategy [73,75,88,100,111]. Most of weather monitoring projects follow the World Meteorology Organization (WMO) standard to set up their network [82,83].

### 5.3. Data Communication Approaches

Efficient communication needs to be established between the sensing and control layer as well as between control and backhaul layer for transmission of sensed data. The advancement of the wireless communication technology has brought significant breakthroughs in IoT. Radio frequency identifiers (RFID) and short-range wireless communications have Bluetooth and ad hoc WSNs as examples. Though these technologies are known to have low power requirements and can operate for a considerable amount of time, most also have short-range coverage and are not suitable for wide spatial coverage applications such as agriculture [129]. Cellular wireless networks can be used to address this limitation but as IoT keeps growing and more objects become connected, network traffic management and signal generation becomes a concern and requires further design improvements [130,131].

A recent communication technology that tries to overcome these limitations is the Low-Power Wide Area Network (LPWAN). It elevates the short-range and high cost of predecessor technology and can connect a wide domain of IoT applications. LPWAN is suitable for services with infrequent and small data exchange requirements. However, it also spans wide spatial coverage with tolerable data loss, making it appropriate for applications that target resource-constrained and remote locations. Table 2 gives examples of wireless communication technology mostly used in IoT.

**Figure 6:**
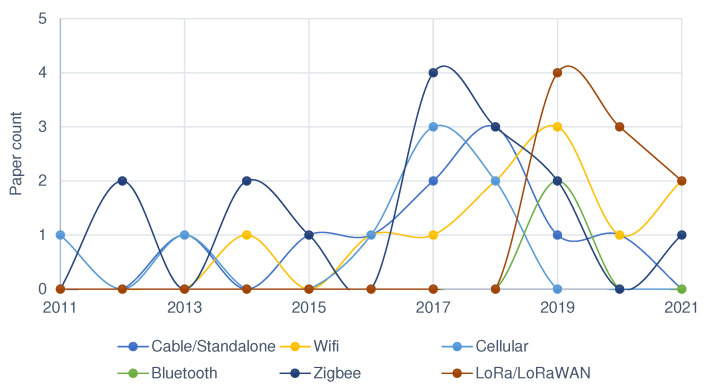
Wireless communication technology preference trends of the reviewed works for smallholder agriculture applications. The Y–axis denotes number of works using a specific communication technology. Some papers have used multiple of this technology, simultaneously.

Preference trends for wireless communication technology in smallholder agriculture over the past ten years are shown in Figure 6. As can be seen, Zigbee and WiFi communication protocols were predominantly used until recently, while LoRa communication has received more interest in the last three years. Both Zigbee and WiFi have limited range coverage but better data rates, making them suitable for short- or medium-distance applications with high data needs. The low duty cycle of the Zigbee protocol, which is an operational method of reducing power consumption, and its large network size also made it preferred in most smallholder agriculture applications [132,133]. A number of projects also operate in standalone mode, a set-up in which a device acts both as a sensing node and as controller or gateway.

**Figure 7:**
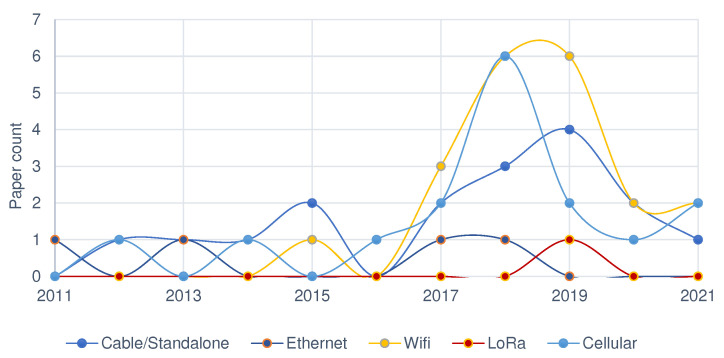
Trends of common backhaul communication standards use in smallholder agriculture applications.

Considering the backhaul communication that allows gateways to transfer received data to application servers, not many alternatives exist in rural areas to cellular communication. The penetration of this technology has increased in developing countries reaching rural areas [134,135,136]. Most publications use the technology for data transmission from farms to central server. Trends in technology choice among the reviewed work is shown in Figure 7. Despite wide penetration, cellular communication is frequently unstable and unreliable in remote areas and may result in data loss. As a result, more reliable options need to be considered.

The choice of an efficient distribution and communication channel among sensing nodes is critical to achieve energy efficiency and reliably collect data. It ensures that nodes have increased lifetime and regular responsiveness. To this end, a WSN mesh topology is common among projects; a few mention the use of star topology [100,113] or a clustered hierarchical topology [110]. In mesh network topology, nodes have routing capability and communication can be established among each other. This allows reliable data transmission and reduced data loss. It requires more power energy and thus suitable for short range communication. In star topology, nodes are connected to one central node with routing capability, and make a star-like structure. Child nodes transmit data to this central hub with no guarantee of delivery. The communication range between nodes and the sink shall thus be kept reasonable or data could be lost. Clustered topology is a tree-like arrangement of two or more star topology to enhance this communication range. The scale, deployment environment and resource have a play on which topology to adopt.

### 5.4. Energy Sources and Saving

**Figure 8:**
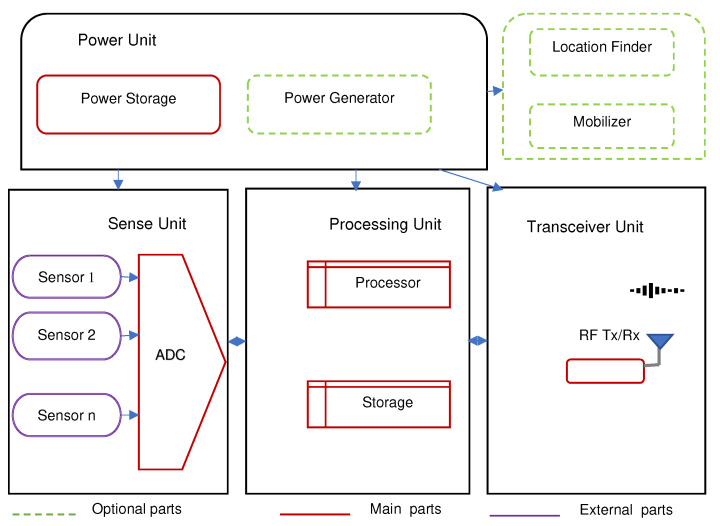
Elementary components of typical sensor node in a WSN (ADC: Analog-Digital-Converter; RF: Radio Frequency; Tx/Rx: Transmitter/Receiver).

Another design consideration for IoT and WSN applications in smallholder agriculture is the power consumption of devices as commonly there is limited or no electricity access. A typical sensing node functional set-up is shown in Figure 8: it has a radio transceiver, a micro-controller board that interfaces with sensors, and a power unit. Dependent on the application, a node can also have extra elements such as a power generator, mobilizer or location finder [137].

Nodes can run on electricity mains, battery, or solar power. For nodes at the sensing layer, easily replaceable Li-Ion batteries are preferred power sources because they are affordable and bring no technical complications. However, frequent replacement is required and caution must be taken on the reliability or quality of transmitted data due to their limited lifetime. A work-around is to apply an energy saving scheme such as sleep-and-awake [76,81,83]. This allows reducing power consumption of nodes in idle state and thus extend battery life. Solar power is also a promising option though it has seasonal fluctuations. Nodes at the control layer need to run uninterrupted and require continuous, reliable power supply. It also demands significant power, which cannot be achieved with simple batteries. The wired electric mains is a sound source for such requirements. It is, however, not always available and then solar panels [66,91,92,106,118,138], rechargeable batteries [81,84,93,94] or power banks [101] are the possible options.

### 5.5. Computational Analysis

**Figure 9:**
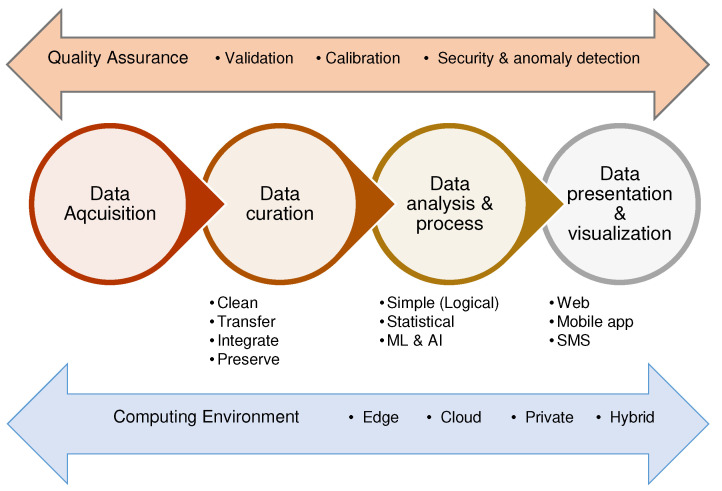
A general dataflow schema in data science computations.

For a systematic study and comparison of approaches taken in the reviewed projects, we used an idealized workflow of a general data science project as presented in Figure 9. It depicts a functional action chain from data acquisition to decision-making and information delivery. It shows how data are produced, processed and presented (PPP) to stakeholders at different operational levels.

- **Data acquisition** This entails the process of what and how data are being collected. In IoT and WSN set-ups, the data acquisition layer focuses on parameter identification, device selection and set-up, communication network selection and establishment, and the data collection phase itself. The sensing and control layers discussed in Section 5 are mapped to this stage of data science.
- **Data curation** In this phase, data cleaning and pre-processing of acquired sensor data are conducted to improve its reliability. Important tasks are filtering, adding, dropping and transferring. It also handles integration of data from different sensors, through well-defined data mapping algorithms. The heterogeneity of devices, operating platforms and absence of de facto communication standards in IoT and WSN communication often obstruct direct and full utilization of the technology. The syntactic and semantic variations at hardware and software levels of the system pose interoperability and integration challenges [93]. Variation in data collection and representation, different communication specifications such as transmission rate and bandwidth, and data processing and presentation are issues to be addressed in this context. One of the critical functionality of a data curation is, thus, facilitation of such data integration. This phase is crucial as the remaining action chains are dependent on its outcome.
- **Data processing and analysis** The data processing and analysis phase is where computations are executed on the raw, sensor data acquired from farms. These computations can be classified into three general groups: (a) Simple, which is a threshold-based if-then analysis that determines incidents as deviations from pre-defined values; (b) Statistical, that determines standard statistics such as regressions and ANOVA; and (c) AI, which brings forecasting and prediction capabilities through advanced mathematical and machine learning computations, using artificial neural network (ANN), deep learning (DL) and other techniques. Operations executed in this phase are based on specific application needs and shall assist the decision-making of smallholder farms. It is responsible to produce usable knowledge from the pre-processed data passed to it.
- **Information presentation and visualization** The presentation and visualization phase handles the human–computer interaction (HCI) and defines appropriate information delivery mechanisms. It also handles the information presentation format prescribed as suitable by the end user. Three broad categories for presentation and data visualization are: web-based, application-based, and SMS- or alert-based. Web-based data presentation can reach users through a stand-alone web page or as social media feeds, such as tweets. The application-based information presentation uses a dedicated mobile application developed and installed on the user’s smartphone. Both web- and application-based mechanisms support graphical and textual data visualization options and provide multi-language access. They do require regular internet access and smart devices. The SMS information presentation is a text-based data delivery that runs on regular phones and can present short alert information to users on the farm status and actions advised.
- **Computing environment** This environment determines the computing capabilities used in all stages of data processing. Edge computing, cloud computing or private computing environments can be used in agriculture data processing. The choice of platform depends on the application needs and resource availability. In an IoT and WSN set-up for agriculture data processing, edge computing can be ideal to process and disseminate information to farmers in real-time. This is also advantageous especially when network communications are fragile, as can be the case in smallholder farming. Edge computing, however, may present challenges in deployment in resource-constrained devices such as sensor nodes and may set limits to data processing implementations. AI algorithms specifically need substantial computation, memory and power resources, which are usually scarce in a WSN. Edge computing also sets higher quality requirements to the software as it is harder to upgrade once deployed.
  Cloud computing offers rich resources to implement sophisticated and large data processing algorithms and persistent storage. It also facilitates the re-usability of open cloud solutions provided as software as a service (SaaS) or infrastructure as a service (IaaS). However, accessibility and network bandwidth demand is a concern in smallholder communities. A private server can be set up with equivalent resource provision to a cloud computing with self-built data analysis and presentation mechanisms.
- **Quality assurance** These are associated tasks that ensure overall data processing and information generation are of some expected quality standard and fit with user expectations. It is a rigorous task that needs to be present in every stage of data processing. During data acquisition, some QA measures can be device testing and calibration, and communication network reliability and efficiency validation. QA during the data curation and data processing phases can be validation of data by pre-processing algorithms in terms of reliability, efficiency and optimality. A dearth of accurate baseline data regarding different agriculture parameters challenges these tasks. Regardless, some quality assurance have to be in place to ensure sensor data are reliable before further investigation and decision support information generation. The computing environment also needs to meet required quality standards such as security, reliability and accessibility.

The quality assurance and computing environments are cross-cutting the action chain of data processing. We believe that such an idealized action chain can serve as a good comparison mechanism between experiments.

We reviewed the publications against these actions and environments of an idealized data science workflow. Table 3 provides an overview of how the methods used align with the schematic actions described above.

### 5.6. Quality Assurance

The calibration of sensors is a critical step before actual deployment in agricultural applications.Laboratory analysis, manual investigation and existing repositories can serve as calibration value sources [76,87,94]. Often, up-to-date ground truth data for most smallholder agriculture is not available, thus requiring alternatives. One alternative can be to compare redundant readings of a sensor and formulation of a calibration value. In these cases, appropriate testing and validation measures must be taken [76,79,82,83]. Outdoor IoT-WSN applications to smallholder agriculture also need to address environmental effects caused by topography, climate and vegetation cover on data quality.

Another critical point related to quality assurance is security and anomaly control. Security is a challenge in IoT and WSN deployment in agriculture as it is distributed through the ecosystem and could happen at different levels. The fact that smallholder farms are found in uncontrolled and often inaccessible areas and the limited literacy rates exacerbate this problem. Security can be perceived from two broad perspectives: external and internal. The external security threats are posed by outside factors such as physical device damage or vandalism, denial of service (DoS) attacks, signal jamming and data fabrications. Internal security threats are anomalies that happen due to wrong data and processing step which compromise the reliability and quality of information generated. Faults in data acquiring and processing anomalies can be mentioned as internal security challenges. Security breach could also happen at device, communication or transaction level. Physical and logical failures of nodes, calibration problem, device’s lifetime and deployment environment could cause anomaly in the sensed data. Communication anomaly could happen due to weak security enforcement, resource or bandwidth competitions among nodes and noise attenuation on the signal transported. The inability to withstand harsh and resource-deprived environment could also challenge the communication standard and result in anomalous data. Ambient temperature and rain pose physical damage risks to sensing nodes and may affect data and signal quality; tree and crop canopy may compromise the communication between sensors and gateway. Lack of proper data curation, and appropriate processing model and algorithm could potentially lead to transaction anomaly and generate unreliable information. All these security problems pose quality concerns to the IoT and WSN generated data which inhibit its utilization to decision supports. Careful consideration of such factors is required before the IoT and WSN deployment for agricultural applications. Waterproof and heat-resistant cases for nodes, integration of external, omnidirectional antennas and/or placement of nodes at appropriate height, utilization of appropriate software tools are relevant design and implementation considerations.

Proper monitoring and analysis schemes may determine the efficiency and reliability of the installed technology. Moreover, appropriate anomaly detection and prevention algorithms need to be deployed at the different layers of the IoT WSN communication to act accordingly and avoid wrong input to a decision making. Some WSN communication standards such as Zigbee and LoRaWAN has added security layer to the protocol to withstand the communication security challenge [27]. Transactions can also be validated at application layer as presented in [62]. These authors have used the block chain technology to enforce security measures on sensor generated data at the application layer. Recent developments in computation platforms such as cloud, edge and fog computing can also be utilized to add security layers to an IoT system. Hardware, software and data protection schemes can be realized at these platforms. Susceptibility of data to interference and other security threats are likely to be reduced if data are processed near the device as in edge or fog computing. These innovations can also enhance energy efficiency of nodes and reduce bandwidth competition between signals and more. These anomaly detection can be executed either at an end node or gateway level. Each end node can be set to monitor all sensors connected to it and detect anomalous properties, in real-time. However, the limited processing and storage capacity of these nodes might inhibit executing resource-intensive and precise anomaly detection algorithms. End node anomaly detection are also unable to identify communication anomalies that could possibly happen at the network layer. A rather optimal anomaly identification can be executed at the gateway node level. As discussed in Section 5, gateway nodes have more storage and computational power and can serve as a more reliable and robust security enforcement platform. In addition to its resource abundance, the gateway node can also be set as a centralized anomaly detection point for both device and communication level anomalies. Such computation capability is well-suited to most smallholder agriculture applications as it eliminates the cloud communication and computation needed for further decisions. The fact that measures can be taken in (near) real-time and using the already established wireless communication makes fog computing at the gateway level more promising. For a bigger WSN setup with several and heterogeneous sub-networks and multiple gateways, a more comprehensive centralized anomaly detection can be carried at higher level such as in cloud computing. Advanced analytical and problem identification algorithms can also run efficiently on such platforms. A hybrid edge and cloud computing based anomaly detection in WSN is presented in [140].

## 6. Discussion

**Figure 10:**
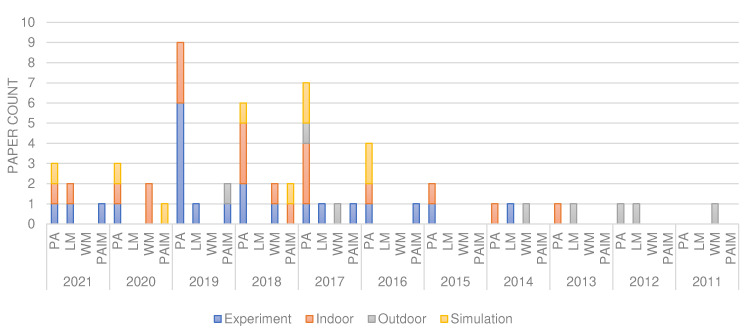
IoT and WSN domains and deployment environment in smallholder agriculture researches (PA:Precision Agriculture, LM: Livestock Management, WM: Weather Monitoring, PAIM: Pest and Animal Infestation Monitoring). The Y-axis labels denote works under each deployment environment and application domain.

In our analysis, we found a significant growth in research effort in IoT and WSN applications for smallholder agriculture over the last decade, with speed-up in the years 2017–2019. Not much of this work has been deployed in farm plots. Figure 10 shows distribution of works in smallholder agricultural application domains on different WSN deployment environment over the last decade. Most work was conducted only experimentally and much remains to be done in actual deployment setting.

Advances in hardware, software and communication technology as well as increased equipment availability are reasons for the increased research interest. In real and outdoor deployment, the gateway and sensing nodes need to be dispersed, especially when a wider spatial coverage is needed. Sensing nodes shall be placed at the farm without risk of loosing of transmission. Additionally, gateway placement should consider stable power provision and a trustworthy communication channel. In doing so, range and network lifetime maximization shall be given utmost considerations. We found no projects that addressed these requirements over a wide time span. Moreover, data and system validation appear not to receive much attention. Long-term behavior of deployed systems and sensed data quality must be evaluated repeatedly to demonstrate system reliability and usefulness. This will establish confidence in any decision-making that utilizes the generated information. Only a few of the projects were found to have longer implementation and validation periods [26,73,79,82,83,88,93]. The remainder were pilot studies or proofs of concept with a short experimentation period of maximally a month.

**Figure 11:**
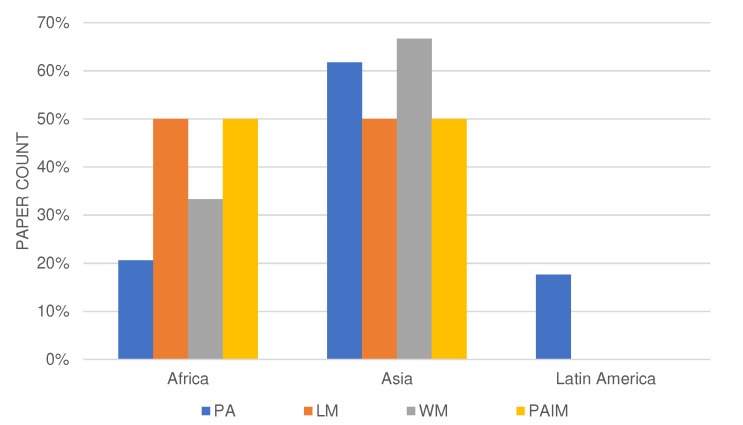
Spatial distribution to continent of IoT and WSN applications in smallholder agriculture (PA:Precision Agriculture, LM: Livestock Management, WM: Weather Monitoring, PAIM: Pest and Animal Infestation Monitoring). The Y-axis denotes percentage distribution of works in the three continents and over each application domain.

**Figure 12:**
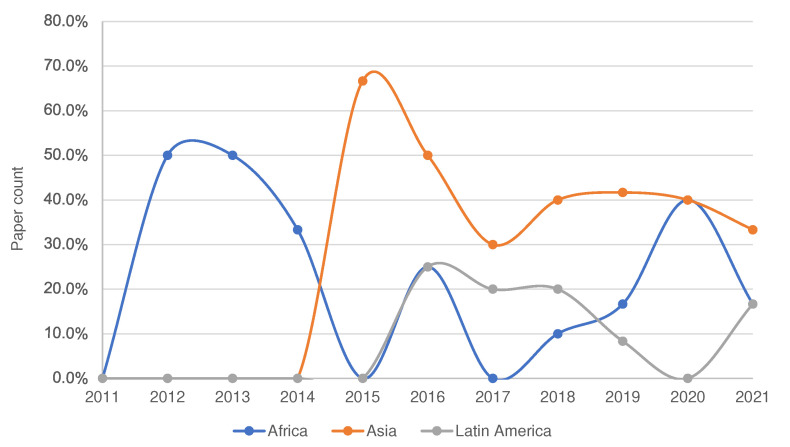
Temporal distribution of IoT and WSN applications in smallholder agriculture. The Y-axis values indicate percentage distribution of works targeting each continent in the specified time period.

Figure 11 and Figure 12 present the reviewed papers’ spatial and temporal distribution over the years of interest and by continent, respectively. In both figures, the Y axis denotes the percentage distribution of projects from the total 53 reviews.

Looking into the continental distribution of projects, Asia is the front-runner (Figure 11) and this aligns with the global Information Communication Technology (ICT) development index report of the International Telecommunication Unit (ITU) [141]. Asian countries are reported to have better ICT coverage and utilization, also in rural areas. This implies that active research is less challenged and equipment may be more readily available. Nonetheless, research in Africa also shows promise. An interesting factor is the heterogeneity of research on the African continent. There seems to be an equivalent effort in all types of smallholder application, possibly due to equivalent importance and impact on farm productivity. We also observed, only 29% of the total projects in Africa are deployed in real-field while 50% and 21% are experimental and simulation executed, respectively. This is also similar in Asia but Latin America has better real-field execution.

As discussed in Section 5.1, PA is researched most widely and usually for the purpose of irrigation management. Smallholder farms apply, however, mostly rain-fed practices with limited or minimal irrigation and so there is potential mismatch between farmer information needs and produced information. The ubiquity of PA and irrigation management may imply two things. First, WSN and IoT implementation and management is easy in a confined area, as in plot-level deployment. It can provide precise data readings with a small number of sensors and few controlling nodes. Secondly, the application will not fully utilize the data provided by the technology. Further research on advanced data analysis is needed as all four domains may have significant effects towards sustainable agriculture.

Even though irrigation is important for sustainable and productive farming, it is not always a solution to the poor productivity of these farmers. Instead, research must focus more on other key parameters such as soil nutrient monitoring or early draught prediction. Such can address the urgently needed data by smallholder farms and assist sustained and efficient utilization of those. Soil moisture data, for instance, can be used beyond irrigation decisions such as in pest management, decisions in appropriate sowing time and crop type to plant [98,120,121]. Information from weather sensors may help farmers to take appropriate measures in situations like drought.

Existing projects utilize off-the-shelf equipment. Furthermore, in smallholder farmer community, technical maintenance and operation skills may not suffice. In dense deployment schemes, the equipment costs may also become a sustenance bottleneck. Such conditions could affect farmer interest to use the technology. Hence, it is important to consider alternatives like producing custom-made devices by integrating components that are open source, simple, affordable and easily accessible. Non-proprietary Arduino or Raspberry Pi (RPi) based node micro-controllers can fit to this purpose. In addition to being open source, these hardware allows researchers to experiment with and program for a fit to the specific needs. A serious online community support also adds to the advantage of using open source devices. Table 4 shows micro-controllers used among reviewed projects. As can be seen in the table, most of the works used Arduino and RPi while in rare cases others like Atmega, MICA and Waspmote are mentioned. Proprietary hardware comes with pre-installed firmware that avoids programming and other technical skills at the expense of flexibility and ease of customization.

Through open source devices and in collaboration with local start-ups, vocational schools or universities, the required equipment can be produced locally, may help to minimize the needed budget and technical assistance. Training and empowering members of the community through hands-on and easily understandable materials, following participatory research where community members are included from conception to execution and working with active projects in the area could also be possible alternatives.

Our review brings to light that Zigbee (35%) and WiFi (31%) WSN communication standards are common in smallholder agriculture. As shown in Figure 10, most work is in indoor or experimental simulations, and this explains the preference for these standards. The significant set-up complexity and cost, limited area coverage and network instability in Zigbee and WiFi makes them less-fitting to smallholder agriculture deployments. In Figure 6, we show that recent experiments work with other WSN communication technologies, such as the Long range (LoRa) LPWAN standard. This indicates future possibilities and rooms for emerging WSN standards to be of use in the agriculture domain.

For gateway–server communication, about 80% of the reviewed work used cellular communication while wired approach is used in rest. They are legitimate choices for the smallholder environment though cellular communication is often unreliable. An alternative is to use a micro-SD in the gateway for storage and later reading, to eliminate data loss as well as transmission mismatches. Edge computing (data processing at the control layer) is a recent technology that shall also be considered when communication between gateway and server is challenged [142]. Communication standards used for node–gateway and gateway–server among the reviewed works is presented in Table 5.

Most reviewed papers focused on establishing the WSN and on data acquisition. The other computing actions defined in Section 5.5 were discussed sparsely, as shown in Table 3. Specifically, the data curation and quality assurance phases, which are crucial for reliable information generation, were not specially described in these works. Neither was much reported on usability of the collected data or how such was used in real application analysis. In the few papers that reported on data analysis, approximately half used simple data analysis, and a quarter statistical and AI analysis. The typical analysis method reported was the determination of a threshold value. For instance, to set an irrigation trigger, a pre-defined soil water content level is checked against the sensor-derived soil moisture level. Or such could trigger an alert message regarding possible crop disease that reaches farmers when the pre-set alert condition is met [60]. Although these approaches are simple and require few computing resources, more precise and robust applications require soft-coded implementations based on holistic and exhaustive scenarios that are hard to handle with pre-sets. Proactive applications of such type can also support reliable prediction and forecasting, which is needed in many agriculture applications [23]. These mechanisms allow farmers to acquire information ahead of time and be aware of what to anticipate on. We postulate they may even come with a learning effect. This in turn will prepare farmers for risks and spur them to explore alternative coping mechanisms.

Real-time farm monitoring also needs to reflect the spatio-temporal dynamics that exist on the farm and that can only be met with advanced computational algorithms, such as using AI. The work by [100] is a good example in this context. The authors developed an agricultural prediction system, capable of generating future complications based on sensor-derived time-series data processed with the auto-regressive integrated moving average (ARIMA) model. This model deploys probabilistic analysis of time-series properties of parameters of interest. The forecasted information is verified against pre-loaded information to generate decision support information. Threshold values change on the basis of forecasts, and when the farmer makes adjustments such lead to periodical updates of thresholds. New computing paradigms such as integration of AI and IoT (AIoT) are expected to revolutionize the data acquisition and processing in IoT applications. Future research shall assess how such innovations can be effectively utilized in agriculture domain.

Among the reviewed works, 31 papers reported on their computing environment with 70%, 21% and 9% in cloud, private and edge computing, respectively. Mostly some cloud environment is used as a persistent data repository and/or data visualization platform. About 30% of the 70% projects involving cloud computing utilized SaaS and IaaS such as The Things Network or Things Speak. Web-based data presentation is the preferred mode in these works (57%), while 26% and 17% utilized mobile applications and SMS data presentation, respectively.

The use of low-cost sensors creates a need to calibrate sensor readings, following specific implementation requirements. Only some projects were found to report on calibration issues [87,94]. Since most projects were experimental, sensors were used as provided by vendors, in off-the-shelf mode. Implementations that use sensor readings for actual decision-making, however, shall take appropriate test and calibration measures before deployment and production. Humans are indispensable in the process of sensor parameter interpretation but none of the projects that we reviewed appeared to have considered the human skills component. We recommend that any platform should integrate humans with sensors for inclusive perception and validation.

We also observed that soil chemical composition and nutrient sensing is almost nonexistent in reviewed work. Research and industry communities need to collaborate to fill such gaps and produce sensors for the unaddressed essential farming factors.

## 7. Challenges

Any innovation or technological intervention comes with challenges and opportunities. Especially when the application is in a remote or resource-constrained location where literacy and awareness are minimal, various limitations may prevent successful implementation. This requires a thorough study and analysis of these factors. The following are some of these challenges that our analysis reveals.

- **High investment costs:** The investments needed to produce and deploy a WSN and IoT system are significant, which may not be affordable by most projects. Hardware costs are those for gateway and sensor nodes, recurring IoT connection, and power provision, while open source tools exist, realistic deployments may need commercial cloud applications such as SAAS, which are a substantial cost factor.
- **Little awareness:** Stakeholders in the agri-chain typically have limited knowledge of recent technological innovations. This is a cause of resistance against acceptance of IoT and sensor technology.
- **Low availability of tools and skilled personnel:** Most IoT and WSN equipment are not produced in the developing countries. Operation and maintenance of devices may require considerable knowledge, language and professional skills. Such are often thin on the ground and this hinders full deployment and utilization.
- **Regulatory and policy gaps:** Telecommunications of many developing world countries have no clear regulatory arrangement of WSN and IoT implementations. The allowed Industrial Scientific and Medical (ISM) radio frequency range for WSN communication is not set yet and no other rules exist governing the use of frequency bandwidth. As a result, investments are risky because node communication may become blocked or interrupted.
- **Poor interoperability:** IoT and WSN have evolved rapidly over the past five years. A number of tools and platforms exist that can be used for the realization of the technology. There is however no standard yet that guarantees their interoperability.
- **Low data quality:** WSN deployment often comes with exposure to extreme physical conditions. This may cause reading faults and poor data quality. Sensor data reliability may also be compromised by deployment errors. Incorrect calibration, inconsistent power supply and unreliable communications can result in data errors such as outliers, drifts and missing values.

## 8. Conclusions

Most smallholder farms are known to be resource-constrained and remotely located which causes sincere challenges in in situ data collection. Consequently, farm-related data are unavailable or is only found in small quantity, often fragmented or outdated. This leads to uninformed farming practices, and consequently poor production and low yields. IoT and WSN may prove to be an assisting tool in addressing information needs. Monitoring crops and soil at farm-scale and in (near) real-time with reasonable cost may help to determine the investments needed to upscale productivity.

The objective of this study is to investigate the potential for actual use of IoT and WSN in more data-intensive approaches to smallholder farm practices. We focus on how the technology can acquire farm-specific and (near) real-time data. The kinds of collected data, the different application domains and the alignment of research needs are also assessed. Design and implementation considerations in smallholder applications are explored as well.

IoT and WSN have been put in practice in large-scale commercial farms but seldomly in smallholder farming, in spite of general agreement on its adaptability with reasonable cost [130,143,144]. Some promising demonstrations of this have been published. Existing works are mostly experimental and may still lack robustness for actual implementation. Actual implementation requires trust in and robustness given that failure may come with unacceptable economic consequences. As a result, investments should be kept in check, and the usability of free and existing communication platforms shall be explored with more case studies and real applications.

Moreover, as most smallholder farms are nestled in poor infrastructural conditions, fit-for-all solutions do not typically work. Future work shall consider the poor infrastructure and limited solution space that needs to be contextualized. The limited or specific parameters being monitored in most existing projects also imply that there is considerable room to integrate additional physical and chemical sensors. They may monitor weather, soil, crop, pests, or provide other data on farm inputs, their integration is essential. WSNs and related technological interventions shall be implemented to set an acceptable standard of smart smallholder agriculture. Diverse and affordable sensors relevant to farm productivity, and applied in mass, may become essential.

Security is a topic mostly overlooked and in its early stage of research in IoT and WSN application to agriculture [145,146]. Particularly, no work is found that addresses this issue among the reviewed projects. The need to integrate various communication standards, devices and data as well as data accuracy and availability need in smart agriculture makes security a challenge. Some of the security issues specific to agriculture and discussed in Section 5.6 exacerbate the severity of the security challenge in this domain. Security threats exist at different levels of the IoT ecosystem and encompass both physical and logical issues. Data integrity, reliability, accuracy and availability are qualities expected of a smart agriculture system. All these create extra demands and opportunities for a rigorous and in-depth researches addressing the matter. The constrained nature of IoT and WSN devices is a setback to practice complex and promising security solutions and this needs to be addressed while proposing security measures.

As most of the reviewed projects are rooted in the prototype stage, no discussion on how such security concerns are addressed is found. Most anomaly detection algorithms rely on some well-defined data and compare current situation against this to detect any deviation or changes. This requires a spatially and temporally detailed and reliable data. However, the absence or sparsity of farm-level information to use as a reference is a concern when it comes to smallholder farms. The fact that anomalies and other security problems potentially inculcate to unreliable decision support system urges for more attention to the domain. Hence, it is necessary to know the existing security vulnerabilities and create mechanisms to mitigate the effects of incidents. Equal emphasis shall be given to security measures as to the deployment of the agriculture system. Selection of appropriate communication standards, layer/s of security measures implementation, level of security need and appropriate security measures identification shall be executed parallel to the system’s functionality development. The usability of existing security and anomaly management techniques for IoT and WSN in agriculture is an open challenge yet to be investigated.

Interoperability and data abstraction are two functionalities a data curation operation in agriculture application needs to offer. Seamless integration of all data coming from various sources and data conversion shall be handled on this phase. Open standards such as the ones by Open Geospatial Consortium (OGC) provide common languages known as sensor model language (SML) to define sensors and their processes, semantically and reduce the syntactic heterogeneity of data representations [147]. Such open standards support efficient machine-to-machine communication and provide seamless integration of heterogeneous devices. It also allows users to interact or access both raw and processed data and reduce latency time. We believe relying on such standard for any WSN setup helps in avoiding and early identification of data anomalies. Such tools shall be explored more and adopted to incorporate these functionality to the data curation phase which is missing in all the reviewed works. The work by [93] is an exception which reports on utilizing the OGC sensor web enablement (SWE) platform for their WSN setup.

Cost and quality are important factors that influence such implementations and projects must focus on affordability and efficiency of technological innovations. The integration of human and electronic sensors can be one way to achieve these requirements. A data acquisition platform that utilizes the most abundantly found and precise sensors within the smallholder community, humans, along with artificial sensors is effective for a whole-sided and inclusive perception. Humans’ read data can also be used to validate the accuracy of sensors’ read data. Geo-referencing sensing nodes is important to obtain location-specific data and monitor farm-level spatial variability which is relevant in most agricultural applications such as PA. Most applications reviewed, however, lack such capability and this remains a research gap to be filled.

One must note the inherent limitations of IoT and WSN, which include limited spatial coverage and data quality. These need to be recognized and addressed. Consequently, there is a need to augment with other data acquisition approaches [63,66,84,148]. Such a data infrastructure must address required spatial and temporal scales. This can be done with complimentary approaches. The use of RS and participatory tools alongside IoT and WSNs is of particular importance [66,149,150].

Citizens’ abundance and capacity to generate as well as validate sensors’ acquired data could play a critical role at all stages of the farm data generation: from collection to logical decision making and interpretations. Human observations can be integrated with WSNs to elevate the limited spatial coverage and enrich the data basket. Some community members can also be used to communicate a rather complex findings to farmers in a simple and usable forms [151]. These open doors to incorporate citizens in any technological interventions to bring about fully-fledged, reliable, sustainable and consumable product.

Soil characteristics specifically are important factors for improved farm yields and are must-have determinants for any intervention. The spatial and temporal variability of soil attributes needs to be understood, both before and during the crop season, to help farmers take appropriate measures. Based on the findings of this paper, the authors are undertaking soil physical and chemical properties monitoring and data collection research through sensors deployment in two districts located in northern Ethiopia. An experimental investigation result of the work is to be communicated soon.

## Figures and Tables

**Table 1:** Systematic search parameters to identify relevant papers for review. * is a wildcard of zero or more search characters and can take ing, er, ers.

Search Parameter	Value
Search query	Internet of Things (IoT) AND (Wireless Sensor Network (WSN) OR sensor)
	AND small AND (agriculture OR farm *) AND “smart farming”
Inclusion criteria	Open access
	Research article, conference proceeding, book chapter, software or data publication
	Small farms or smallholder agriculture
	Wireless communication
	2010–2021
Exclusion criteria	Targets mechanized, large-scale farms, developed countries
	Non-English manuscripts
	Less technical details or mostly higher-level descriptions
	Theoretical frameworks
	Non-agricultural applications
Citation databases	ACM
	Scopus
	ScienceDirect

**Table 2:** Wireless communication technology comparison with example.

Wireless Technology	WiFi	WiMAX	ZigBee	Cellular	Bluetooth	LoRa
Wireless network	WLAN	WMAN	WPAN	WWAN	PAN	LPWAN
Standard	802.11 *	802.16 *	802.15.4 *	2/3/4 G	802.15.1 *	LoRaWAN
Operating Frequency (Hz )	5–60 G	2–6 G	868/919 M	2.4 G, 865 M	2.4 G	433/868/900 M
Data Rate (bps )	1 M–6.75 G	1 M–1 G	40–250 K	50 K–1 G	1–24 M	0.3–50 K
Transmission Range	20–100 m	<50 km	10–20 m	Entire cellular	8–10 m	<50 km
Power Consumption	High	Medium	Low	Medium	Medium	Very low
Cost	High	High	Low	Medium	Low	High
Operating Life	Years	Hours	Up to 2 years	Hours	Hours	10–20 Years
Noise Resistance	Low	Medium	Medium	Medium	Low	High
References	[58,59,60,61,62,63,64,65,66,67,68,69,70]	[71,72]	[62,73,74,75,76,77,78,79,80,81,82,83]	[84,85,86,87,88,89,90,91,92,93,94]	[95,96,97,98,99]	[100,101,102,103,104,105,106]

*: IEEE

**Table 3:** Assessment report of reviewed works based on defined data science actions. Note that categorizations are not exclusive and some works utilized multiple classes of computing action. ISO/IEC 9126 describes software characteristics for quality metrics.

Data Science Action	Techniques/Approaches
Data curation	Data preservation [61,64,74,75,84,85,89,102,105,111,115]
	Data transfer to JSON and XML formats [75,89,93,102]
	Data fuzzification and de-fuzzification [62]
	Redundant data removal [61]
Data analysis and processing	If then [60,64,68,69,85,91,93,99,104,111,115]
	Statistical [61,80,87,88,89,100,105]
	ML and AI [62,63,65,76,92,94,103]
Data presentation and visualization	Web-based [58,61,63,64,74,75,76,81,84,91,92,93,94,99,100,105,111,115,138,139]
	App-based [62,68,69,85,100,102,104,105,139]
	SMS-based [60,85,87,92,104,115]
Computing environment	Cloud [58,60,62,64,65,68,69,75,76,82,85,89,91,93,102,104,105,115,138,139]
	Edge and/or Fog [61,99,103]
	Private server [74,81,84,87,94,100,111]
Quality assurance measures	Non-elaborated calibration [84,93,106]
	Data validation based on descriptive statistics [104]
	Reliability and data accuracy assessment based on ISO/IEC 9126 [83]
	Sensor calibration based on standard laboratory results [76]
	Sensor calibration using conventional weather station readings [94]
	Sensor data validation against standard laboratory results [82]
	Sensor data validation using linear correlation [65]
	Transaction validation based on block chain [62]

**Table 4:** Major micro-controllers used in smallholder agriculture application projects (PA:Precision Agriculture, LM: Livestock Management, WM: Weather Monitoring, PAIM: Pest and Animal Infestation Monitoring).

Micro-Controllers	Application Domain			
	PA	LM	WM	PAIM
Arduino	[62,77,78,85,87,98,100]	[86,104]		[81]
Atmega	[89,91,96,102]	[79]		[91,92]
NodeMCU	[58,68]		[70]	[60,61]
RPi	[63,64,84,101,139]		[83,105]	
Others	[73,74,75,76,110,113]		[82,94]	

**Table 5:** WSN and backend communication standards adopted in smallholder agriculture applications (PA:Precision Agriculture, LM: Livestock Management, WM: Weather Monitoring, PAIM: Pest and Animal Infestation Monitoring).

Network	Communication Standard	Application Domain			
		PA	LM	PAIM	WM
WSN	Bluetooth	[95,96,97,98]		[99]	
	GPRS/GSM	[84,85]	[86]		
	LoRa/LoRaWAN	[100,101,102,103]	[104]		[105]
	WiFi	[58,59,89,98]		[60,61]	[93]
	Zigbee	[62,73,74,75,76,77,78,88,106]	[79,80]	[81]	[82,83]
	Wired	[63,64,65,68,96,113]		[92]	[70,94]
Backhaul	GPRS/GSM	[73,77,87,88,89,90,102]	[86,104]	[91,92]	[93,94]
	LoRa	[106]			
	WiFi	[58,62,63,64,65,66,67,68,69,85,89,97,98]		[60,61,99]	[70,82]
	Ethernet/standalone	[74,78,84,95,96,100,103,110,113,139]	[79]	[81]	[83,105]
